# Phenotypic Screening of H_1_-Antihistamines Identifies Promethazine and Rupatadine as Active Compounds Against *Toxocara canis* Infective Larvae

**DOI:** 10.3390/ph18070997

**Published:** 2025-07-02

**Authors:** Taís C. Silva, Julia Godoy-Silva, Monique C. Amaro, João V. Silva-Silva, Thiago H. Doring, Leonardo L. G. Ferreira, Adriano D. Andricopulo, Josué de Moraes

**Affiliations:** 1 Research Center on Neglected Diseases, Guarulhos University, Guarulhos 07023-070, SP, Brazil; taisnith@hotmail.com (T.C.S.); jugodoy.silva@outlook.com (J.G.-S.); moniqueamaronpdn@gmail.com (M.C.A.); 2 Laboratory of Medicinal and Computational Chemistry (LQMC), Institute of Physics of Sao Carlos (IFSC), University of Sao Paulo (USP), Sao Carlos 13563-120, SP, Brazil; jvssilva89@gmail.com (J.V.S.-S.); thiago.doring@ufsc.br (T.H.D.); leonardo@ifsc.usp.br (L.L.G.F.); 3 Department of Exact Sciences and Education (CEE), School of Technology, Exact Sciences and Education (CTE), Federal University of Santa Catarina (UFSC), Blumenau 89036-256, SC, Brazil; 4 Research Center on Neglected Diseases, Scientific and Technological Institute, Brazil University, São Paulo 08230-030, SP, Brazil

**Keywords:** *Toxocara canis*, H1 antihistamine, β-tubulin, density functional theory, molecular docking

## Abstract

**Background**: Parasitic worm infections remain among the most prevalent and neglected health issues worldwide, affecting both humans and animals. Toxocariasis, caused by *Toxocara* spp., is a widespread zoonosis with significant public health and economic implications. Current anthelmintic treatments show limited efficacy, particularly against tissue-migrating larvae, underscoring the urgent need for new therapeutic options. This study aimed to evaluate the anthelmintic potential of H_1_ antihistamines as repurposed drug candidates against *Toxocara canis*. **Methods**: Twenty-two H_1_ antihistamines were screened for larvicidal activity against infective third-stage (L3) larvae of *T. canis*. Larval motility and morphology were assessed, and compounds with the highest efficacy were further investigated using density functional theory (DFT) to explore their electronic properties. Molecular docking simulations were also performed to predict interactions with *T. canis* β-tubulin. **Results**: Promethazine and rupatadine exhibited significant larvicidal effects, surpassing albendazole in reducing larval motility and inducing a distinct contorted morphology not observed in control or albendazole-treated larvae. DFT analyses suggested a strong electron-acceptor capacity, indicating a potential redox-based mechanism of action. Docking studies revealed favorable binding to the colchicine site of *T. canis* β-tubulin. **Conclusions**: This is the first report of larvicidal activity of antihistamines against *T. canis*, supporting their potential as repurposed therapeutic agents for the treatment of zoonotic helminthiases, particularly those caused by tissue-migrating nematodes.

## 1. Introduction

Parasitic helminth infections remain a major global health burden, affecting millions of humans and animals annually [1,2]. Among these, toxocariasis, caused by the nematode *Toxocara canis*, is one of the most prevalent zoonotic diseases worldwide [3]. This parasite is transmitted primarily through the ingestion of infective eggs from contaminated soil, water, or food, with dogs and other canids serving as definitive hosts [4]. In humans, *T. canis* larvae migrate through tissues, causing visceral larva migrans, ocular larva migrans, and covert toxocariasis, which can lead to chronic inflammation, organ damage, and neurological complications [3,5]. Despite its significant public health and economic impact, toxocariasis remains a neglected tropical disease with limited treatment options [6].

Global seroprevalence studies reveal that toxocariasis is widely distributed across continents, with high infection rates particularly in low- and middle-income countries, where poor sanitation, limited access to veterinary care, and high environmental contamination facilitate transmission [7]. These findings highlight the urgent need for effective control measures and novel therapeutic interventions.

The primary treatment for toxocariasis relies on anthelmintic drugs, with albendazole as the first-choice therapy and mebendazole as the second-line option. However, their efficacy is often suboptimal, requiring prolonged treatment courses and showing variable success in eliminating larval stages [1,4]. Moreover, the emergence of drug resistance in parasitic nematodes underscores the need for novel therapeutic agents with improved efficacy and safety profiles [8]. In alignment with the United Nations Sustainable Development Goals (SDGs), the World Health Organization (WHO) has emphasized the importance of investing in the research and development of new anthelmintic drugs. This approach aims to reduce the burden of neglected tropical diseases (NTDs), improve global health equity, and support sustainable healthcare systems in endemic regions [9].

Recent advances in the molecular biology of helminths have opened new avenues for drug discovery. Although albendazole and mebendazole remain standard therapies, their limitations have prompted the search for novel targets. Neurotransmitter receptors have emerged as attractive candidates, given their role in parasite motility, feeding, and survival [10,11]. Among these, histamine receptors have been identified in various helminth species and are involved in neuromuscular regulation [12]. Histamine, a biogenic amine, acts through receptors that are expressed in the nervous and muscular systems of parasitic nematodes [13,14].

In light of these findings, H_1_-antihistamines have emerged as attractive candidates for drug repurposing in helminth infections. In addition to their well-established clinical safety, many of these compounds exhibit polypharmacological profiles—interacting with neuromuscular and cholinergic targets beyond histamine receptors—which may enhance their antiparasitic activity. In vitro studies have already demonstrated the efficacy of certain H_1_-antihistamines against phylogenetically diverse helminths, including *Schistosoma mansoni* [15,16], and *Angiostrongylus cantonensis*, reinforcing their potential as broad-spectrum agents. Despite these promising indications, their activity against *T. canis* larvae remains largely unexplored.

**Figure 1:**
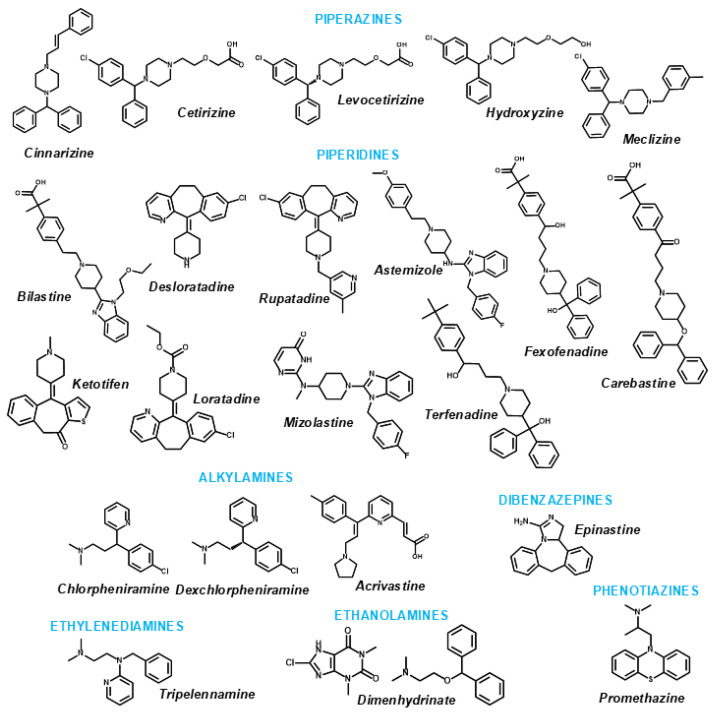
Chemical structures of H_1_ antihistamines.

In this study, we investigated the anthelmintic activity of 22 antihistaminic H_1_ drugs (Figure 1) against infective larvae (L3) of *T. canis*. We aimed to identify compounds that could impair larval motility and viability, as well as induce morphological changes, as indicators of anthelmintic efficacy. Additionally, density functional theory (DFT) calculations were used to evaluate the electronic properties of the compounds, providing insights into their reactivity and stability. Molecular docking simulations were also performed to explore potential molecular interactions and support the interpretation of in vitro findings.

## 2. Results

**Figure 2:**
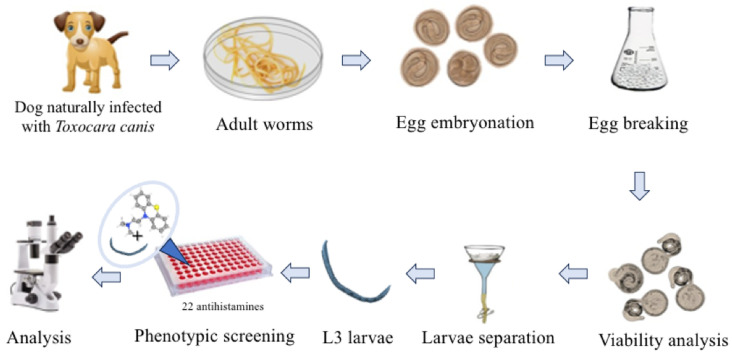
Experimental workflow for evaluating antihistamine activity against *Toxocara canis* L3 larvae. Adult worms were collected from naturally infected dogs, and their eggs were isolated and embryonated to the infective larval stage. A total of 22 antihistamines were screened, leading to the selection of promethazine and rupatadine for EC_50_ determination.

The main experimental workflow of this study is summarized in Figure 2. Adult *T. canis* worms were collected from naturally infected dogs, and their eggs were isolated and embryonated to the infective third larval stage (L3). A phenotypic screening of 22 antihistamine drugs was performed against L3 larvae. Among the compounds tested, promethazine and rupatadine demonstrated significant anthelmintic activity at a concentration of 50 µM. These two drugs, along with the reference anthelmintic albendazole, were selected for further evaluation using serial dilutions to determine their half-maximal effective concentrations (EC_50_).

**Figure 3:**
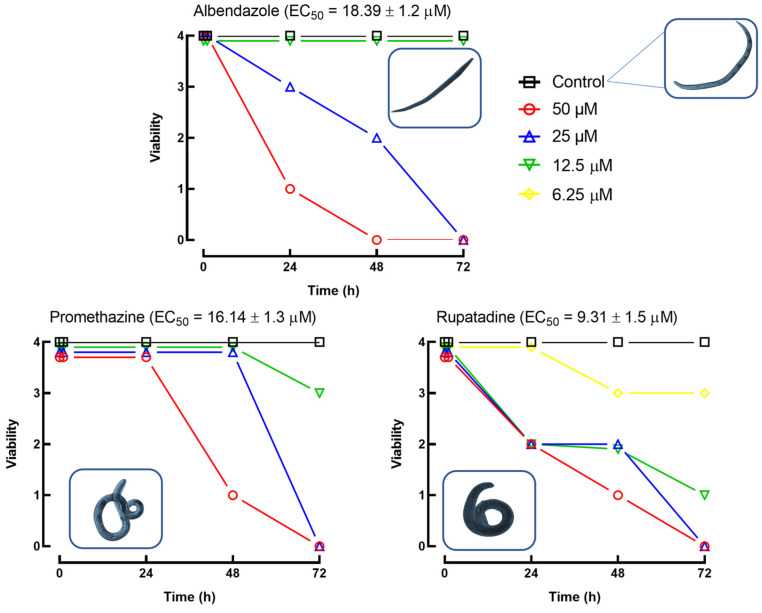
Time- and concentration-dependent effects of albendazole, promethazine, and rupatadine on the viability of *Toxocara canis* L3 larvae. Larval motility was scored on a scale from 4 (highly active) to 0 (dead), and the number of larvae in each category was recorded at different time points. Values represent the total counts across three independent wells per condition. EC_50_ values were calculated based on the proportion of larvae reaching immobility (score 1), using nonlinear regression from triplicate datasets, and are presented with standard deviation (±SD). Among the tested compounds, rupatadine exhibited the highest potency, followed by promethazine and albendazole (*p* < 0.05). Representative light microscopy images of treated and untreated larvae are presented alongside each viability curve.

The time- and concentration-dependent effects of albendazole, promethazine, and rupatadine on L3 larval viability are shown in Figure 3. Viability was scored on a scale from 4 (fully active) to 0 (dead). At 50 µM, all three compounds induced complete immobility (score 1) within 24 h and lethality (score 0) by 48 h. Despite this similar temporal pattern, the compounds exhibited distinct potencies and effects at lower concentrations.

Albendazole had the fastest onset of action, yet it was the least potent, with no significant effect at or below 12 µM and an EC_50_ of 18.39 µM. Promethazine displayed intermediate potency (EC_50_ = 14.14 µM), slightly reducing motility (score 3) at 12.5 µM after 72 h, with no observable effect at 6.25 µM. Rupatadine was the most potent compound (EC_50_ = 9.31 µM), significantly impairing larval viability at 12.5 µM (score 2 at 24 h, score 1 at 72 h) and still inducing reduced motility (score 3) at 6.25 µM as early as 24 h. These differences in EC_50_ values were statistically significant (*p* < 0.05), highlighting distinct potencies among the tested compounds.

In addition to viability scoring, light microscopy was used to assess morphological alterations in larvae exposed to the compounds. Larvae treated with promethazine or rupatadine exhibited a characteristic contorted morphology, primarily affecting the posterior region of the body. This phenotype was not observed in larvae exposed to albendazole, which retained an elongated shape similar to that of untreated controls. These morphological findings suggest a distinct neuromuscular effect induced by promethazine and rupatadine that is absent with albendazole.

**Figure 4:**
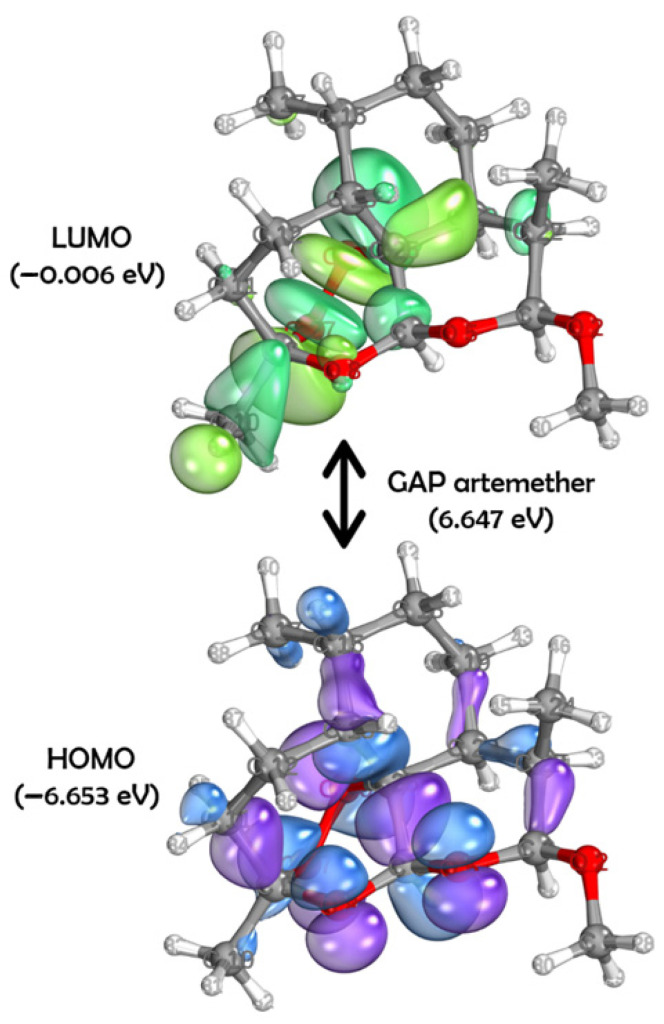
Surfaces of HOMO and LUMO to artemether ((1R,4S,5R,8S,9R,10S,12R,13R)-10-methoxy-1,5,9-trimethyl-11,14,15,16-tetraoxatetracyclo [10.3.1.04,13.08,13]hexadecane), obtained through the DFT method using the set of basis functions def2-TZVPP and functional B3LYP. For HOMO: in purple, positive phases; in blue, negative phases. For LUMO: In dark green, positive phases; in light green: negative phases.

To explore the electronic and structural properties potentially related to the anthelmintic activity of the compounds, quantum chemical calculations were performed. According to the data presented in Figure 4, artemether has a HOMO energy of −6.653 eV and a LUMO energy of −0.006 eV, resulting in an energy gap (GAP) of 6.647 eV. The HOMO and LUMO molecular orbital surfaces, obtained through DFT, reveal the primary atomic orbital contributions. For the HOMO, the main contributions include: S on atoms C3, C6, C9, C11, C20, and C25; Py on atoms C6, O5, O8, C25, O26, and O27; Px on atoms C6, O26, and O27; and Pz on atom O26. For the LUMO, orbital contributions were observed as: S on atoms C10, C22, H31, O26, O27, and H7; Pz on atoms C6, C15, C20, and O27; Px on atoms O26 and O27; and Py on atoms C6, C25, and O26.

In artemether’s HOMO, positive phase sigma bonding was observed between C3–C22, C19–C20, C9–C11, and C6–C25, while negative phase sigma bonding occurred between C20–C22, C15–H16, and C17–H39. For the LUMO, an antibonding orbital interaction was noted between atoms O26 and O27. In both orbitals, the electron density was primarily concentrated in the oxygen-containing rings.

**Figure 5:**
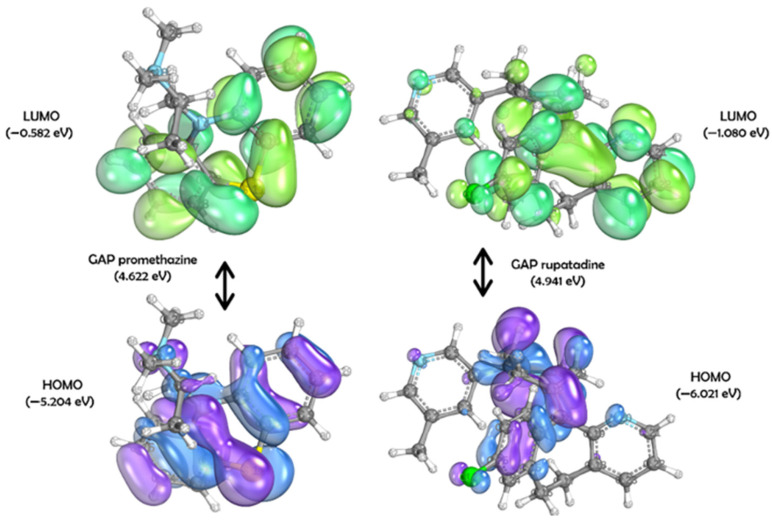
On the left: surfaces of HOMO and LUMO to promethazine (N,N-dimethyl-1-phenothiazin-10-ylpropan-2-amine). On the right: surfaces of HOMO and LUMO to rupatadine (13-chloro-2-[1-[(5-methylpyridin-3-yl)methyl]piperidin-4-ylidene]-4-azatricyclo[9.4.0.03,8]pentadeca-1(11),3(8),4,6,12,14-hexaene). Both obtained through the DFT method using the set of basis functions def2-TZVPP and functional B3LYP. For HOMO: in purple, positive phases; in blue, negative phases. For LUMO: in dark green, positive phases; in light green: negative phases.

As shown in Figure 5, promethazine and rupatadine displayed smaller energy gaps: 4.622 eV and 4.941 eV, respectively. Promethazine has HOMO = −5.204 eV and LUMO = −0.582 eV, while rupatadine has HOMO = −6.021 eV and LUMO = −1.080 eV. For promethazine’s HOMO, Py contributions were observed on atoms N4, C5, C10, S11, and C12, with additional S, Px, and Pz contributions on N4. In the LUMO, Px contributions were seen on C5, C7, S11, and C12; Pz on C10 and C17; S on C5, C10, C12, and C17; and Py on C5, C7, C10, C12, and C15.

Promethazine’s HOMO featured a delocalized system across C10–C5–C6 and C12–C17–C16, with delocalized electron density in the sulfur-containing central ring. Additional π-bond interactions occurred between C7–C8 and C14–C15, while σ-bond interactions were found between C3–C2 and C3–H25. In the LUMO, primary π-bonding interactions included C8–C9, C10–S11, S11–C12, and C13–C14. In both orbitals, electron density was primarily located in the fused tricyclic ring system.

Similarly, Rupatadine’s HOMO showed S and Py contributions mainly from atom N9, while its LUMO displayed S contributions on C11, C16, C29, and H42; Pz on C12, C22, and C23; Px on C14 and C19; and Py on C13. Notable π-bonding interactions were found between C12–C13 and C25–C26 in the HOMO, and between C24–C15 in the LUMO. A delocalized π-system extended over C14–C13–C19, along with a π lone pair on C27. As with promethazine, both frontier orbitals in rupatadine were largely centered in the fused tricyclic region.

To gain further insights into the interaction of promethazine and rupatadine with potential molecular targets, we modeled the β-tubulin protein from *T. canis* for molecular docking studies. The β-tubulin sequence (KHN79367.1) was retrieved from NCBI and aligned via BLASTP against the PDB. Using the predefined selection criteria, PDB 5GON was chosen as the template. The sequence similarity between *T. canis* tubulin and the template chain 5gon.1.B was 95.13%, supporting its suitability for homology modeling.

A high-quality model was generated using SWISS-MODEL, yielding a GMQE score of 0.82 and a QMEANDisCo Global score of 0.80 ± 0.05, indicating strong similarity to the target structure. The model, based on a beta-lactam bridged analogue-tubulin complex, also received favorable validation metrics: a ProSA-Web Z-score of −9.98 and a MolProbity score of 1.48, with a low clash score (1.2) and 96.95% of residues in favorable regions of the Ramachandran plot. These metrics confirm the model’s suitability for docking studies (detailed in Supplementary Materials).

To validate the docking protocol, the co-crystallized ligand from the template structure (compound 6ZR, a β-lactam analogue of combretastatin A-4) was redocked into the active site of the *T. canis* β-tubulin model. The redocked ligand displayed a root-mean-square deviation (RMSD) of 0.3908 Å compared to the original position (Table S1), indicating high accuracy. Key interactions were maintained, confirming the structural reliability of the homology model and the docking approach.

**Figure 6:**
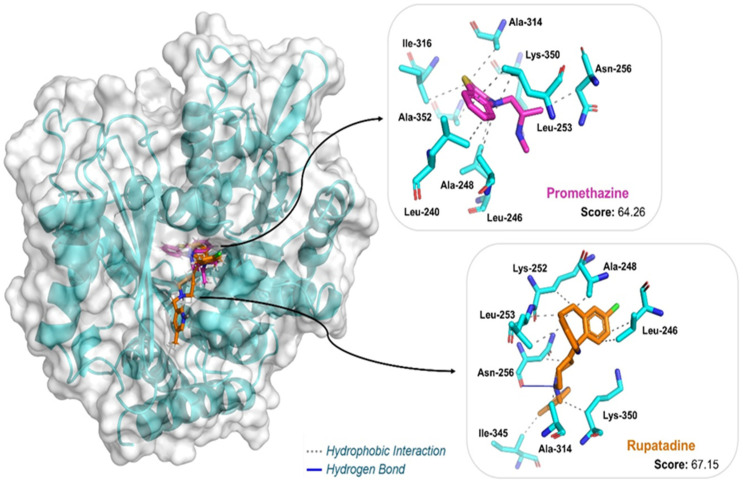
Three-dimensional view of the molecular docking show binding interactions of H1-antihistamine drugs in the *T. canis* β-tubulin model. The figure was generated using PyMol v3.0.3 (Schrödinger, LLC, New York, NY, USA).

Molecular docking simulations showed that both promethazine and rupatadine interacted favorably with the colchicine-binding site of the modeled *T. canis* β-tubulin (Table 1, Figure 6). Rupatadine exhibited the highest docking score (67.15), followed by promethazine (64.26). Both ligands were mainly stabilized by hydrophobic interactions with conserved residues such as Leu246, Ala248, Leu253, Ala314, and Lys350, consistent with colchicine-site ligands.

Promethazine formed hydrophobic contacts with nine residues, including Leu240 (3.44 Å), Leu246 (3.43 Å), and Ala248 (3.10 Å). Rupatadine engaged similar residues and additionally established a hydrogen bond with Asn256 (3.23 Å), possibly contributing to its higher binding affinity.

## 3. Discussion

This study identified promethazine and rupatadine as antihistamine drugs with significant in vitro anthelmintic activity against *T. canis* infective larvae. At a concentration of 50 µM, both compounds induced pronounced larval immobility and death, with rupatadine exhibiting the greatest potency (EC_50_ = 9.31 µM), followed by promethazine and albendazole. Although albendazole showed the fastest onset of action, its efficacy decreased at lower concentrations, consistent with clinical observations of limited activity against tissue-migrating larval stages [1,4].

Light microscopy revealed that larvae treated with promethazine or rupatadine developed a characteristic contorted morphology, especially affecting the posterior region. In contrast, larvae treated with albendazole or left untreated retained a straight, elongated shape. This phenotype, commonly associated with neuromuscular dysfunction or sustained spastic paralysis, suggests that promethazine and rupatadine may act via mechanisms distinct from those of albendazole, which primarily targets microtubule dynamics [1]. These observations align with previous findings by our group showing consistent anthelmintic activity of antihistamines against the flatworm *S. mansoni* and the nematode *Angiostrongylus cantonensis*, with promethazine demonstrating broad efficacy across species [12,16]. Notably, the anthelmintic activity of antihistamines appears to be independent of their H_1_-receptor affinity, indicating alternative or additional mechanisms of action.

The selection of H_1_-antihistamines for this screening was guided by their established clinical safety and growing evidence of antiparasitic effects in various helminth species [12,15,16]. These compounds represent chemically diverse subclasses, including tricyclic phenothiazines (e.g., promethazine) and piperidines (e.g., rupatadine), each with distinct pharmacological properties that may contribute to anthelmintic activity [17]. Promethazine has demonstrated broad-spectrum effects in prior studies with *S. mansoni* and *A. cantonensis*, potentially due to its anticholinergic and membrane-disruptive actions [12,15,16]. Rupatadine, although structurally distinct, exhibits dual H_1_ and platelet-activating factor (PAF) receptor antagonism, along with anti-inflammatory effects. These features may enhance its therapeutic potential, particularly in mitigating tissue damage during larval migration [18]. Together, these characteristics justify the inclusion of H_1_-antihistamines in phenotypic drug discovery pipelines for helminthiases, including toxocariasis.

An important methodological consideration is the use of serum-free RPMI 1640 medium in our in vitro assays. This choice was based on established protocols for *T. canis* larvae, which remain viable and motile for several days under these conditions. Additionally, excluding serum minimizes pharmacological interference, as serum proteins can sequester test compounds and affect their availability. However, a recent study demonstrated that serum supplementation can significantly influence drug responses in *Haemonchus contortus*, a blood-feeding nematode of small ruminants [19]. Although species-specific physiological differences should be considered, these findings underscore the need to optimize in vitro culture systems to better reflect in vivo environments.

A critical aspect of anthelmintic drug development is the elucidation of the mechanisms by which larval impairment is achieved [20]. This not only informs structure–activity relationships but also guides the rational design of more effective compounds and therapeutic strategies.

The observed neuromuscular effects—especially rapid paralysis and the contorted larval morphology—point toward possible interaction with cholinergic pathways. Unlike classical anthelmintics that target nicotinic acetylcholine receptors, antihistamines may act on muscarinic-like receptors, such as GAR-3, which are conserved in nematodes [12]. Promethazine’s known anticholinergic activity supports this hypothesis, suggesting a biphasic neuromuscular disruption that warrants further exploration.

These findings are further supported by the theoretical framework of density functional theory (DFT), a widely used method to investigate the electronic properties of bioactive molecules in relation to their biological activity [21,22,23,24]. By analyzing the energies of molecular orbitals, such as E_HOMO_ and E_LUMO_, and the electron density distribution in compounds, it is possible to gain insights into how electronic interactions affect the ability to inhibit parasites [25].

In general, the HOMO energy serves as a more reliable indicator of antioxidant activity. On the other hand, LUMO energy is more relevant for assessing antiparasitic activity, as it reflects the molecule’s tendency to undergo reduction [25,26]. E_HOMO_ measures the electron-donating character of a compound (potential reducing agent), while E_LUMO_ indicates the electron-accepting character (potential oxidizing agent) [27].

Oxidative stress caused by *T. canis* has been widely explored in the literature [28]. Elgendy et al. (2024) [29] reported increased oxidative markers in the liver of infected animals, which were only partially reversed by artemether—a drug with known pro-oxidant and antiparasitic effects [29,30,31]. Promethazine and rupatadine showed greater electron-accepting capacity than artemether, as demonstrated in Figure 5. Similar oxidative effects have been described for other anthelmintics, including albendazole, whose primary mechanism involves β-tubulin binding but which can also induce reactive oxygen species (ROS) generation [32].

In addition to the redox-related effects, molecular docking simulations demonstrated that both promethazine and rupatadine interact favorably with the colchicine-binding site of the *T. canis* β-tubulin homology model. The docking poses revealed hydrophobic stabilization by conserved residues such as Leu246, Ala248, Leu253, Ala314, and Lys350—similar to those involved in the binding of combretastatin analogues [33], which are established colchicine-site ligands. While these findings are consistent with known interactions at this site, they provide only preliminary in silico support for β-tubulin as a potential secondary target, complementing the known neuromuscular and redox-based actions of these drugs.

In summary, promethazine and rupatadine emerge as promising, fast-acting anthelmintic candidates for the treatment of toxocariasis. Their larvicidal activity appears to involve multiple mechanisms, including neuromuscular disruption, oxidative stress induction, and possible interaction with β-tubulin, as suggested by computational analysis. Further experimental studies are required to validate these proposed mechanisms of action, particularly the involvement of β-tubulin and nematode muscarinic receptors. Given their availability and safety, these antihistamines represent a viable repurposing opportunity for toxocariasis and other helminthiases caused by tissue-migrating larvae.

## 4. Materials and Methods

### 4.1. Drugs and Reagents

A total of 22 antihistamine drugs were evaluated in this study: cinnarizine, chlorpheniramine, deschlorpheniramine, dimenhydrinate, promethazine, ketotifen, meclizine, tripelennamine, acrivastine, astemizole, bilastine, carebastine, cetirizine, desloratadine, fexofenadine, levocetirizine, loratadine, misolastine, epinastine, hydroxyzine, rupatadine, and terfenadine. These compounds were purchased from Sigma-Aldrich (St. Louis, MO, USA), Toronto Research Chemicals (Toronto, ON, Canada), and Cayman Chemical (Ann Arbor, MI, USA). All compounds were obtained in analytical grade with purity ≥ 98%, as reported by the respective manufacturers. RPMI 1640 culture medium, penicillin G sulfate, and streptomycin were obtained from Vitrocell (Campinas, SP, Brazil). HEPES buffer, glutaraldehyde solution, and dimethyl sulfoxide (DMSO) were purchased from Sigma-Aldrich (St. Louis, MO, USA).

### 4.2. Parasite Collection and Preparation

Adult female *Toxocara canis* worms were collected from naturally infected dogs. Eggs were extracted from the uteri of the worms and incubated in 2% formalin solution for 30 days at 28 °C with >90% humidity to induce embryogenesis. The protein coat of the eggs was removed using a 6% sodium hypochlorite solution at 35 °C. After incubation, the eggs were washed in phosphate-buffered saline (PBS, pH 7.2). Eggshells were mechanically disrupted using glass beads [34]. The contents of the eggs were then subjected to the Baermann method to isolate active larvae, which were used in subsequent in vitro assays.

### 4.3. In Vitro Assays with Toxocara canis L3 Larvae

In vitro assays were performed using *T. canis* third-stage (L3) larvae. Prior to testing, larvae were washed in RPMI 1640 medium supplemented with 200 U/mL penicillin and 200 µg/mL streptomycin. They were then transferred to 96-well plates at a density of 100 larvae per well, each well containing containing RPMI 1640 medium supplemented with 25 mM HEPES and the same antibiotics [35].

For the initial screening, larvae were exposed to each antihistamine compound at a concentration of 50 µM. Each treatment was performed in triplicate (n = 3 independent wells per compound). Compounds showing significant activity were subsequently tested at lower concentrations for dose-response evaluation. For these assays, three independent experiments were performed, and in each experiment, all treatment conditions were tested in triplicate. Albendazole served as the positive control, while wells containing only the culture medium were used as negative controls [36].

Larval behavior was monitored under a microscope or stereomicroscope over a 72 h period, with observations recorded every 24 h. Motility and mortality were used as the primary evaluation parameters. Larval motility was scored based on a five-point scale: 4 (highly active, full-body movement), 3 (slow movement involving the entire body), 2 (movement restricted to a single body region), 1 (immotile but viable), and 0 (dead), following the criteria established by [35]. Larval mortality was confirmed using 0.4% Trypan Blue staining.

### 4.4. Statistical Analyses

All statistical analyses were conducted using GraphPad Prism version 8.0 (GraphPad Software, San Diego, CA, USA). Half-maximal effective concentrations (EC_50_) were calculated based on the proportion of larvae reaching immobility (score 1), using nonlinear regression with sigmoidal dose–response (variable slope) models [37]. These values were obtained from three independent experiments, each performed in triplicate, and are reported as mean ± standard deviation (SD). Group comparisons were performed using one-way ANOVA followed by Tukey’s multiple comparisons test. Differences were considered statistically significant at *p* < 0.05.

### 4.5. DFT Studies

The 2D structure of the compounds were generated using ChemSketch 2021.1.1 and converted into a single SMILES database file. Initial geometry optimization was carried out in Avogadro (1.2.0 version) [38], using Ghemical Force Field with convergence set to ΔE < 1 × 10^−3^ kJ mol^−1^. Quantum chemical calculations (DFT) were then conducted in the gas phase to estimate energy values. Highest occupied molecular orbital (HOMO), lowest unoccupied molecular orbital (LUMO), and gap (LUMO-HOMO) were calculated on ORCA 5.0.2 [39] using B3LYP [40] functional and def2-TZVPP (Weigend and Ahlrichs, 2005) basis sets. The molecular orbitals are generated on IBOView (v20211019) [41].

### 4.6. Homology Modeling

β-Tubulin has been explored to explain the mechanism of action of anti-helmintics in nematodes [1]. To support this investigation, several amino acid sequences of *Toxocara canis* β-tubulin were retrieved from the UniProt database (https://www.uniprot.org (accessed on 12 April 2025)). Due to the presence of different isoforms or variants of β-tubulin, a correlation step was necessary to identify the most relevant sequence for this study. The sequence corresponding to accession number KHN79367.1 was selected based on its correlation with results from a BLASTp search against the Protein Data Bank proteins (pdb) database in the National Center for Biotechnology Information (NCBI) server (http://blast.ncbi.nlm.nih.gov/Blast.cgi (accessed on 19 April 2025)). Sequences with >90% identity, >90% coverage, and E-value < 10^−4^ were selected, leading to the identification of PDB 5GON as the most suitable structural template for further analysis. The sequence similarity between 5gon.1.B and KHN79367.1 was assessed using Clustal Omega (https://www.ebi.ac.uk/Tools/msa/clustalo/ (accessed on 24 April 2025)). The 3D structure of KHN79367.1 was modeled using SWISS-MODEL (https://swissmodel.expasy.org/ (accessed on 24 April 2025)) [42,43,44,45] and validated through Ramachandran plot statistics, rotamer outliers, and clash scores using MolProbity analysis [46,47,48]. Model quality was further assessed using GMQE, QMEAN values [49], and ProSA-Web for overall quality and Z-scores [50].

### 4.7. Molecular Docking

Promethazine and rupatadine were drawn in MarvinSketch 24.3.0 (ChemAxon) with pH adjustments reflecting physiological conditions for β-tubulin [51]. The structures were energy-minimized in Avogadro v1.2.0 using the MMFF94 force field (dE = 1 × 10^−7^ kJ/mol) and exported as MOL2 files [38]. Molecular docking was performed using a homology-modeled structure of *T. canis* β-tubulin, with surface charge distribution calculated at physiological pH [51] using APBS and PDB2PQR servers (http://www.Poissonboltzmann.org/, accessed on 3 May 2025) with the PARSE force field [52]. The docking procedure used ChemPLP (with ASP as rescore), performing 10 independent runs per structure. A root mean square deviation (RMSD) of less than 2.0 Å was used as the criterion for successful predictions. The binding site was defined by the geometric center of the co-crystallized ligand in the 5GON template, with a 10 Å spherical grid. The results were analyzed with the PLIP web server [53] to identify protein–ligand interactions and visualized using PyMOL v3.0.3 (Schrödinger, LLC).

## 5. Conclusions

Promethazine and rupatadine demonstrated potent in vitro activity against *T. canis* larvae, surpassing albendazole in efficacy at lower concentrations and inducing a distinct contorted morphology. DFT analysis revealed that both compounds possess a higher electron-accepting capacity, suggesting a potential pro-oxidant effect. Molecular docking revealed favorable interactions with β-tubulin at the colchicine-binding site. These findings highlight β-tubulin as a potential therapeutic target, although further experimental validation is required to confirm its role and to elucidate the mechanisms underlying the anthelmintic effects of these compounds against *T. canis*.

## Figures and Tables

**Table 1:** Scores of the predicted binding modes of H1-antihistamine drugs after docking in the *T. canis* β-tubulin model, based on the active site of template 5gon.1.B.

Ligand	Docking Score	Interaction	Residues (Distance in Å)
Promethazine	64.26	Hydrophobic	Leu240 (3.44), Leu246 (3.43), Ala248 (3.10), Leu253 (3.52; 3.93), Asn256 (3.60), Ala314 (3.96), Ile316 (3.98), Lys350 (3.37), Ala352 (3.31)
Rupatadine	67.15	Hydrophobic	Leu246 (3.40; 3.48), Ala248 (3.07), Lys252 (3.53), Leu253 (3.43; 3.54), Asn256 (3.80), Ala314 (3.56), Ile345 (3.45), Lys350 (3.91)
		Hydrogen Bond	Asn256 (3.23)

These interactions were calculated with the Protein-Ligand Interaction Profiler (PLIP) web server. Score was determined using ChemPLP (with ASP rescore).

## Data Availability

Data is contained in the paper.

## Supplementary Materials

The following supporting information can be downloaded at https://www.mdpi.com/article/10.3390/ph18070997/s1.
