# Supplementary material for: Phenotypic Screening of H1-Antihistamines Identifies Promethazine and Rupatadine as Active Compounds Against Toxocara canis Infective Larvae
Source: Pharmaceuticals (Basel). 2025 Jul 2;18(7):997. doi: 10.3390/ph18070997 (PMC12297986; doi:10.3390/ph18070997)
Supplement: Supplementary file 1 [file pharmaceuticals-18-00997-s001.zip › pharmaceuticals-3721207-supplementary.pdf]

Phenotypic Screening of H1-Antihistamines Identifies Promethazine and Rupatadine as Active Compounds Against *Toxocara canis* Infective Larvae

Taís C. Silva[1], Julia Godoy-Silva[1], Monique C. Amaro[1], João V. Silva-Silva[2], Thiago H. Döring[2,3], Leonardo L. G. Ferreira[2], Adriano D. Andricopul [2,], Josué de Moraes [1,4,]**

[1] Research Center on Neglected Diseases, Guarulhos University, Guarulhos, SP, 07023-070, Brazil. taisnith@hotmail.com (T.C.S.); jugodoy.silva@outlook.com (J.G.S.); moniqueamaronpdn@gmail.com (M.C.A.).

[2] Laboratory of Medicinal and Computational Chemistry (LQMC), Institute of Physics of Sao Carlos (IFSC), University of Sao Paulo (USP), Sao Carlos. SP, 13563-120, Brazil. jvssilva89@gmail.com (J.V.S.S.); thiago.doring@ufsc.br (T.H.D.); leonardo@ifsc.usp.br (L.L.G.F.); aandrico@ifsc.usp.br (A.D.A.).

[3] Department of Exact Sciences and Education (CEE), School of Technology, Exact Sciences and Education (CTE), Federal University of Santa Catarina (UFSC), Blumenau, SC, 89036-256, Brazil.

[4] Research Center on Neglected Diseases, Scientific and Technological Institute, Brazil University, São Paulo, SP, 08230-030, Brazil.

*Correspondence: josue.moraes@prof.ung.br or moraesnpdn@gmail.com (J.d.M.); aandrico@ifsc.usp.br (A.D.A.)

Suplementary Materials

Table S1. Features of the *Toxocara canis* tubulin model (accession no. KHN79367.1) generated with SWISS-MODEL, using *Bos taurus* tubulin (PDB ID: 5GON) as the reference structure.

Template	5gon.1.B	Resolution	2.48Å
Seq. Identity	95.02	Seq. Similarity	0.61
Oligostate	Monomer	GMQE	0.82
Coverage	0.95	QMEANDisCo Global	0.80 ± 0.05
Found by	user alignment	Description	Tubulin beta chain
Method	X ray		

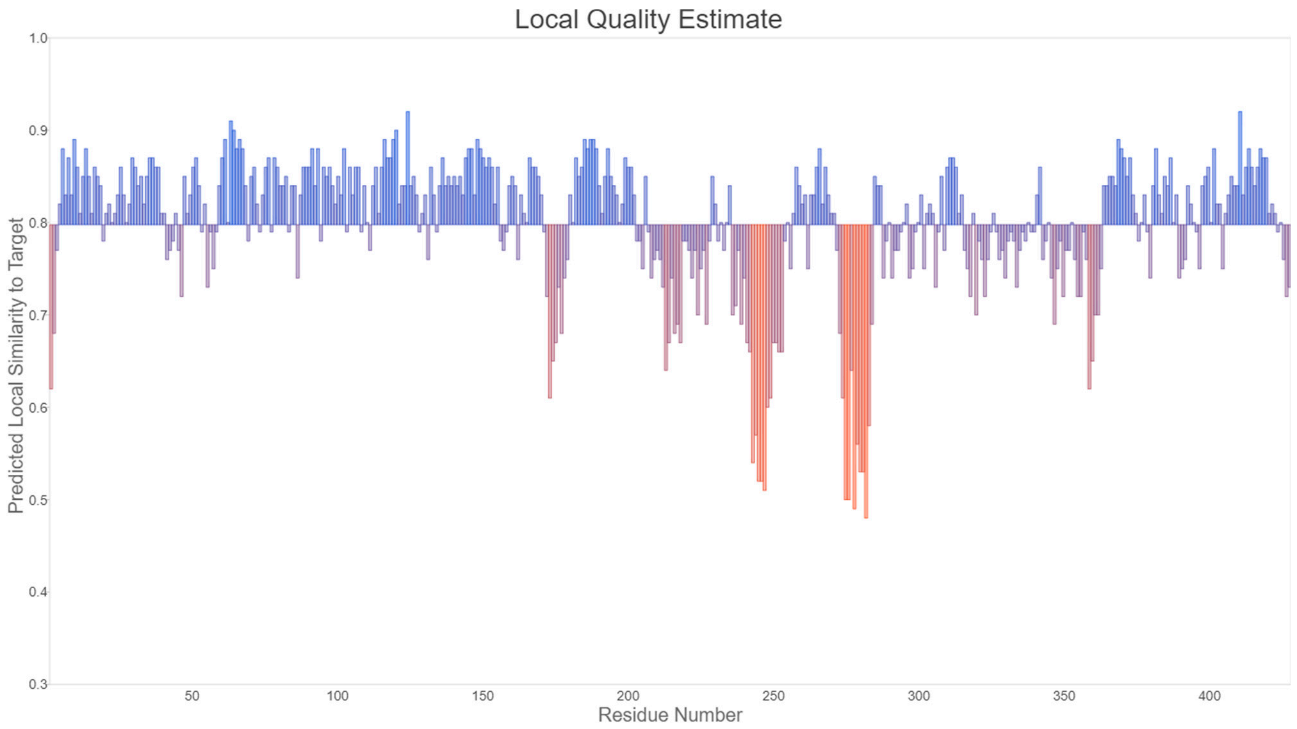

Figure S1. Local quality estimate of the modelled *Toxocara canis* tubulin (accession no. KHN79367.1) obtained from SWISS-MODEL. The quality assessment was performed using QMEANDisCo, incorporating information from the reference structure *Bos taurus* tubulin (PDB ID: 5GON).

```
5gon.1.B        MREIVHIQAGQCGNQIGAKFWEVISDEHGIDPTGSYHGDSDLQLERINVYYNEATGNKYV    60
KHN79367.1      MREIVHIQAGQCGNQIGAKFWEVISDEHGIDPTGAYNGDSDLQLERINVYYNEASGGKYV    60
                **********************************:*:*****************:*.***

5gon.1.B        PRAILVDLEPGTMDSVRSGPFGQIFRPDNFVFGQSGAGNNWAKGHYTEGAELVDSVLDVV    120
KHN79367.1      PRACLVDLEPGTMDSVRAGPFGQLFRPDNFVFGQSGAGNCWAKGHYTEGAELVDNVLDVV    120
                *** *************:*****:*************** **************.*****

5gon.1.B        RKESESCDCLQGFQLTHSLGGGTGSGMGTLLISKIREEYPDRIMNTFSVMPSPKVSDTVV    180
KHN79367.1      RKEAESCDCLQGFQMTHSLGGGTGSGMGTLLISKIREEYPDRIMNTFSVVPSPKVSDTVV    180
                ***:**********:**********************************:**********

5gon.1.B        EPYNATLSVHQLVENTDETYCIDNEALYDICFRTLKLTTPTYGDLNHLVSATMSGVTTCL    240
KHN79367.1      EPYNATLSVHQLVENTDETFCIDNEALYDICFRTLKLTTPTYGDLNHLVSMTMSGVTTCL    240
                *******************:****************************** *********

5gon.1.B        RFPGQLNADLRKLAVNMVPFPRLHFFMPGFAPLTSRGSQQYRALTVPELTQQMFDSKNMM    300
KHN79367.1      RFPGQLNADLRKLAVNMVPFPRLHFFMPGFAPLTSRGSQQYRSLTVPELTQQMFDAKNMM    300
                ******************************************:************:****

5gon.1.B        AACDPRHGRYLTVAAIFRGRMSMKEVDEQMLNVQNKNSSYFVEWIPNNVKTAVCDIPPRG    360
KHN79367.1      AACDPRHGRYLTVAAIFRGRMSMKEVDEQMLNVQNKNSSYFVEWIPNNVKTAVCDIPPRG    360
                ************************************************************

5gon.1.B        LKMSATFIGNSTAIQELFKRISEQFTAMFRRKAFLHWYTGEGMDEMEFTEAESNMNDLVS    420
KHN79367.1      VKMAATFIGNSTAIQELFRRVSEQFTAMFRRKAFLHWYTGEGMDEMEFTEAESNMNDLIS    420
                :**:**************:*:*************************************:*

5gon.1.B        EYQQYQDATADEQGEFEEEEGEDEA       445
KHN79367.1      EYQQYQDATADEEGDFDEHDQEIE-       444
                ************:*:*:*.: * *
```

Figure S2. Multiple sequence alignment between *Toxocara canis* tubulin (accession no. KHN79367.1) and the template 5gon.1.B. Asterisks (*) indicate identical amino acids, colons (:) indicate conserved substitutions, dots (.) indicate semi-conserved substitutions, and dashes (—) represent gaps. The alignment was performed using Clustal Omega (https://www.ebi.ac.uk/jdispatcher/msa/clustalo).

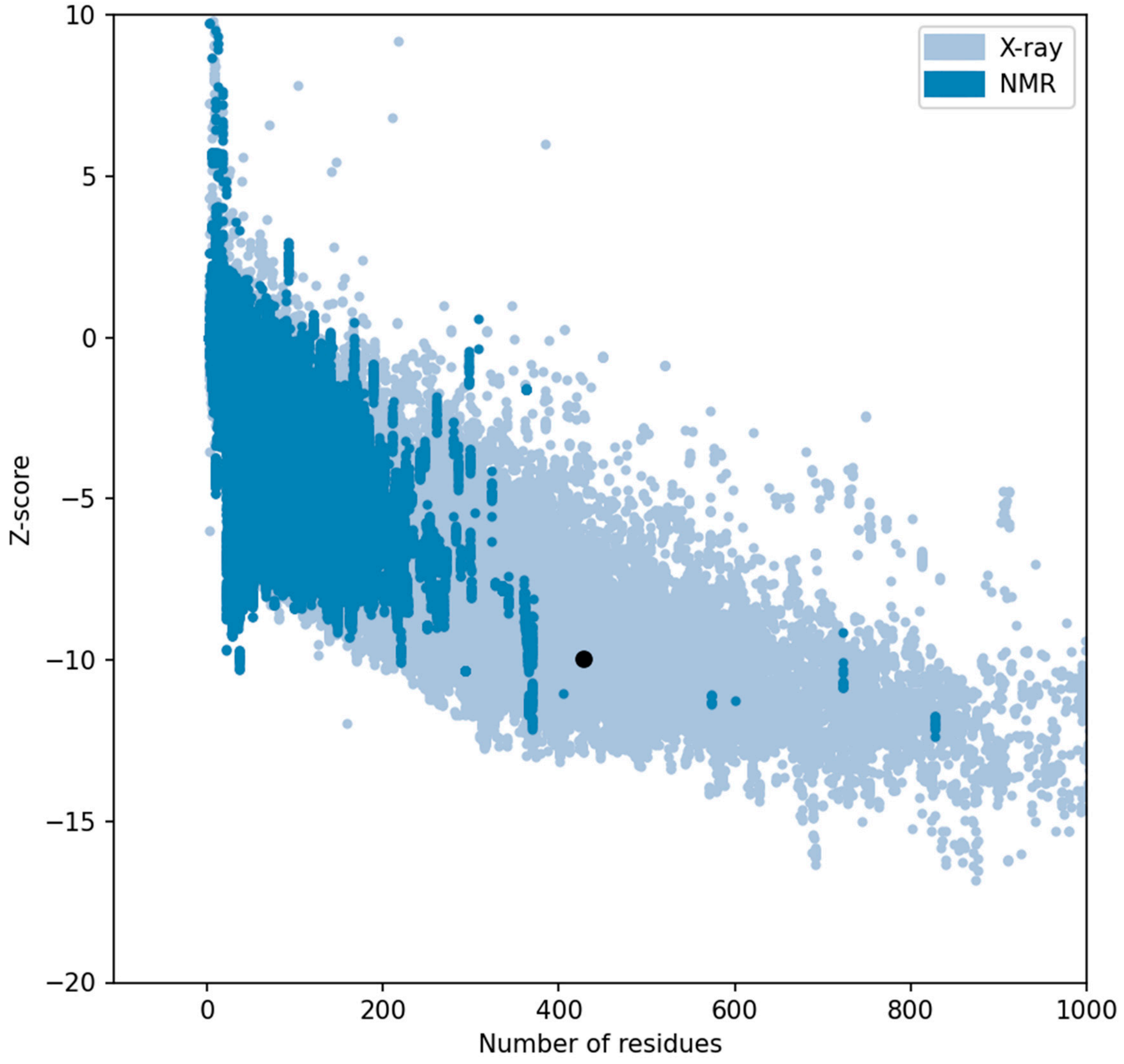

Figure S3. ProSA-Web validation of the *Toxocara canis* tubulin (accession no. KHN79367.1) 3D model generated with SWISS-MODEL. The Z-score (−9.98) falls within the range of high-quality experimental structures, confirming the model's reliability for further functional and biochemical analyses.

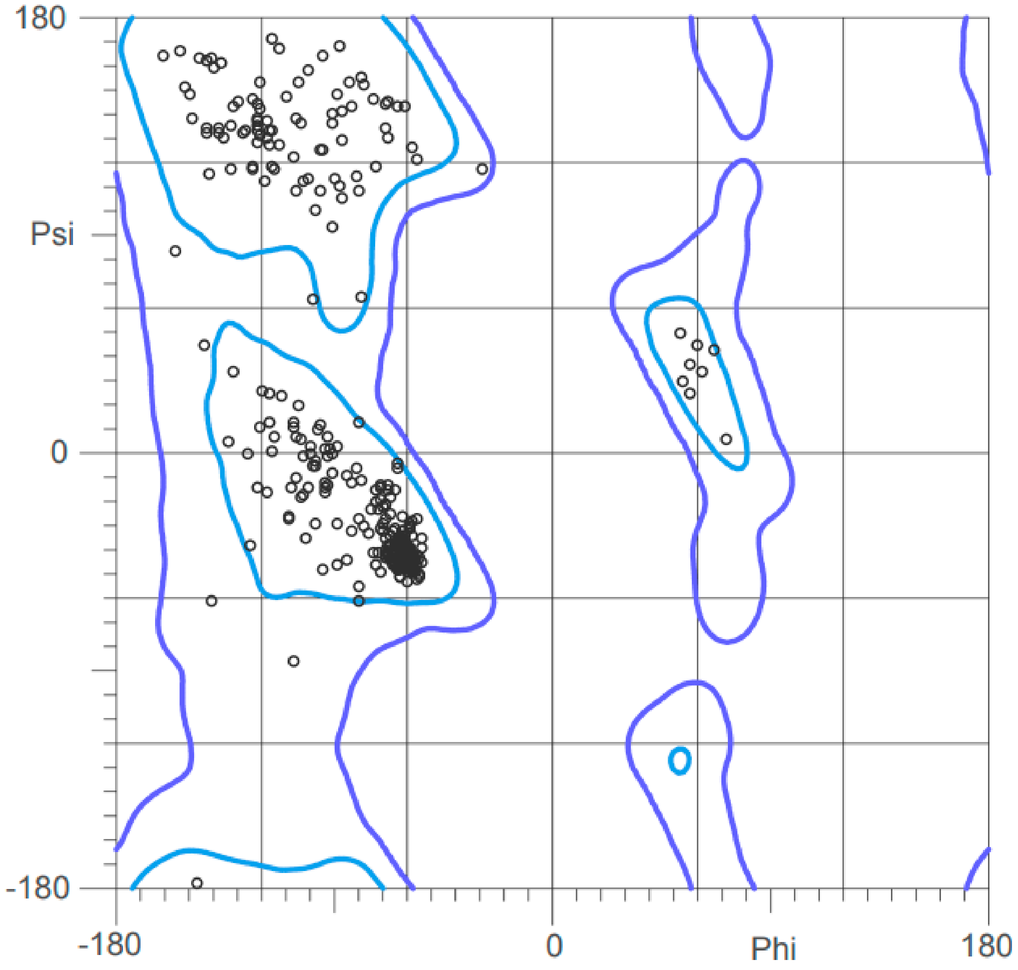

Figure S4. Ramachandran plot analysis of the predicted *Toxocara canis* tubulin (accession no. KHN79367.1) model, showing the distribution of backbone dihedral angles (Phi, Psi). A total of 96.95% of residues fall within the favored regions (blue contours), and 100.0% are in the allowed regions (purple contours), with no outliers detected. These results confirm a well-folded and stereochemically reliable model, supporting its structural accuracy and stability. The analysis was performed using MolProbity.

Table S2. Redocking of the co-crystallized ligand 6ZR ((3R,4R)-4-(4-methoxy-3-oxidanyl-phenyl)-3-methyl-1-(3,4,5-trimethoxyphenyl)azetidin-2-one) into the active site of the β-lactam-bridged analogue bound to tubulin (PDB ID 5gon.1.B) and into the *T. canis* β-tubulin model.

**Target**	**RMSD**	**Docking Score**	**Interaction**	**Residues (Distance in Å)**
5gon.1.B	0.3527	67.90	Hydrofobic	Lys254 (3.98), Leu255 (3.48), Ala316 (3.81), Lys352 (3.60; 3.86)
			Hydrogen Bond	Asp215 (2.98)
Model	0.3908	65.21	Hydrofobic	Leu246 (3.99), Ala248 (3.94), Lys252 (3.90), Leu253 (3.42), Ala314 (3.85), Lys350 (3.51; 3.82)
			Hydrogen Bond	Asp249 (2.96)

Model: generated using SWISS-MODEL based on PDB 5gon.1.B. These interactions were calculated with the Protein-Ligand Interaction Profiler (PLIP) web server. Score was determined by ChemPLP (with ASP rescore).
